# Effect of resveratrol on SNARE proteins expression and insulin resistance in skeletal muscle of diabetic rats

**DOI:** 10.22038/IJBMS.2019.13988

**Published:** 2019-12

**Authors:** Azam Rezaei Farimani, Mohammad Taghi Goodarzi, Massoud Saidijam, Reza Yadegarazari, Sadegh Zarei, Soheila Asadi

**Affiliations:** 1 Noncommunicable Diseases Research Center, Neyshabur University of Medical Sciences, Neyshabur, Iran; 2 Department of Biochemistry, School of Medicine, Hamadan University of Medical Sciences, Hamadan, Iran; 3 Research Center for Molecular Medicine, Hamadan University of Medical Sciences, Hamadan, Iran; 4 Department of Molecular Medicine and Genetics, Medical School, Hamadan University of Medical Sciences, Hamadan, Iran; 5 Department of Clinical Biochemistry, School of Medicine, Rafsanjan University of Medical Sciences, Rafsanjan, Iran; 6 Department of Clinical Biochemistry, Facultyl of Medicine, Kermanshah University of Medical Sciences, Kermanshah, Iran

**Keywords:** Resveratrol, SNAP-23, SNARE complex proteins, Syntaxin-4, Type 2 diabetes, VAMP-2

## Abstract

**Objective(s):**

Soluble N-ethylmaleimide-sensitive factor attachment protein receptor (SNARE) complex proteins are involved in membrane trafficking. The expression of isoforms of SNAP-23, syntaxin-4, and VAMP-2 is significantly done in skeletal muscles; they control GLUT4 trafficking. It is believed that type 2 diabetes could be caused by the modifications in the expression of SNARE complex proteins. The purpose of this study was to evaluate the effect of resveratrol on the expression of these proteins in type 2 diabetes.

**Materials and Methods:**

Forty male Wistar rats were selected. Streptozotocin and nicotinamide were applied for the induction of type 2 diabetes. The animals were divided into five groups. Healthy and diabetic groups were set as control; resveratrol (1, 5, and 10 mg/kg body weight) was applied to treat the three groups of diabetic rats for 30 days. Real-time qRT-PCR was applied to evaluate the expression of SNARE complex proteins.

**Results:**

There is a link between diabetes and insulin resistance and up-regulation of SNARE proteins expression. Resveratrol improved hyperglycemia and insulin resistance along with a non-significant reduction in the expression of SNARE proteins.

**Conclusion:**

Increased expression of SNARE proteins was possibly a compensatory mechanism in response to insulin resistance in the skeletal muscles of diabetic rats. Resveratrol non-significantly reduced the expression of SNARE proteins by enhancing insulin sensitivity, where this effect was dose-dependent. Thus, higher doses of resveratrol and longer intervention periods could probably be more effective. Another molecular mechanism of the anti-diabetic properties of resveratrol was identified with an effect on the expression of SNARE proteins.

## Introduction

Type 2 diabetes mellitus (T2DM) is one of the most common metabolic disorders worldwide associated with severe complications. Insufficient insulin production/secretion by the pancreatic beta cells and insulin resistance are two leading pathological causes of T2DM; therefore, these abnormalities are associated with defects in glucose uptake and glucose homeostasis, which can cause hyperglycemia (1). In insulin-resistant individuals, glucose cannot be transported into skeletal muscle cells and adipocytes. These tissues are two insulin-sensitive organs that use the glucose transporter type 4 (GLUT4) system to remove glucose from the blood. In the basal state, GLUT4 transporter is stored in an intracellular compartment, which quickly lies on the cell surface upon insulin stimulation (2, 3). Due to the central role of GLUT4 in insulin-dependent glucose uptake, impaired GLUT4 translocation can cause insulin resistance (4). Among the regulatory proteins, the soluble N-ethylmaleimide-sensitive factor attachment protein receptor (SNARE) complex proteins play essential roles in trafficking, docking, and fusion with the plasma membrane (5, 6). A ternary complex made up of the v-SNARE protein or vesicle-associated membrane protein (VAMP) interacting with the t-SNARE proteins of syntaxin and synaptosomal-associated protein is formed by SNARE complex (7). Of various isoforms of SNARE proteins, SNAP-23, syntaxin-4, and VAMP-2 are highly expressed in skeletal muscle tissue and control GLUT4 trafficking in muscle cells (8, 9); consequently, dysfunction of this ternary complex and changes in SNARE proteins levels can cause T2DM (10). Previous evidence suggests that intramuscular levels of GLUT4 are normal in T2DM, while its translocation is disrupted (10). Some studies have indicated that insulin exocytosis declines due to decreased expression of SNARE proteins in T2DM (11). Besides, some studies have found that the expression of SNARE proteins can increase in insulin resistance state in muscle tissue (12). Therefore, according to the available evidence, changes of SNARE proteins levels are associated with insulin resistance and diabetes; however, it is unclear whether these changes lead to insulin resistance and hyperglycemia or whether they occur secondary to these conditions. Identification of factors that improve insulin sensitivity and glucose tolerance can help prevent or even treat T2DM. In recent years, researchers have conducted many studies on natural factors that have anti-diabetic effects such as resveratrol (RSV) (13). RSV (3, 5, 4′-trihydroxystilbene), a plant-derived polyphenol, is found profoundly in grapes, red wine, and other plant sources (14). Now, it is made into dietary supplementation tablets. Studies reported that RSV influences insulin secretion and boosts glucose tolerance in animals and humans with T2DM (15). Also, it is known that RSV has anti-hyperglycemic effects and can enhance the expression and translocation of GLUT4 to the plasma membrane (16, 17). The aim of the present study was to show if RSV is capable of influencing the expression of SNARE complex proteins in skeletal muscle cells and look for another molecular mechanism of RSV function in T2DM. For this purpose, an animal model of T2DM was obtained by injecting streptozotocin (STZ) and nicotinamide (NA), which could emulate human T2DM (18).

## Materials and Methods

***Materials***

Streptozotocin, nicotinamide, and insulin ELISA kit were purchased from Sigma Aldrich (Germany). Resveratrol supplements were procured from Amazon (USA), and fasting blood glucose kit was obtained from Pars Azmun (Iran). 

***Animals***

Forty male Wistar rats (8–10 weeks old, mean weight: 200–250 g) were selected from the Razi Institute (Iran). The rats were kept under standard environmental and nutritional conditions during the experimental period (The Central Animal House, Hamadan University of Medical Sciences). Fresh water and standard chow were applied to feed the animals. They were put under a cycle of 12 hr/light: 12 hr/dark. The study protocol was approved by the ethics committee of Hamadan University of Medical Sciences.

***Induction of type 2 diabetes and treatment***

After overnight fasting, the rats were injected intraperitoneally with a single dose of streptozotocin–nicotinamide (STZ-NA) to induce type 2 diabetes. In particular, at first, 60 mg/ kg streptozotocin (0.1 M in sodium citrate; pH, 4.5) was administered. After 15 min, 120 mg/kg nicotinamide (dissolved in normal saline) was injected (19). Citrate buffer was injected alone in the control rats. Then, 72 hr after the injection, diabetes was confirmed by measuring fasting blood glucose (FBS) in tail vein blood using a glucometer (Accu Chek; Roche, Germany) (20). The rats with basal FBS levels above 150 mg/dL were considered diabetic. Treatment began seven days after induction of diabetes for 30 days. RSV (dissolved in deionized water) was administered orally using a gavage syringe (21). Based on randomization, the rats were put into five groups (n=8) as follows: the first and second groups were healthy and untreated diabetic rats, respectively (as controls); the other three groups consisted of diabetic rats that received various doses of RSV for 30 days (1, 5, and 10 mg/kg body weight/day).

***Sample collection***

Following the end of the treatment, the rats were anesthetized and killed by Ketamine: Xylazine (100 mg/kg: 5-10mg/kg IP). The tissue samples were separated from the soleus muscle, placed in cryotubes, quickly frozen in liquid nitrogen, and transferred to a -80 ^°^C freezer. Five-milliliter blood samples were collected (through cardiac puncture) and centrifuged (10 min at 2500 rpm) to separate serum. Then, they were kept at -20 ^°^C.

***Total RNA extraction***

RNA extraction was done by homogenizing frozen tissues (300 mg) in liquid nitrogen with a pestle. Trizol (Invitrogen) was used for manual extraction of RNA (23). 

Thoroughly homogenized tissues were placed in a tube containing 1 ml Trizol and incubated for 10 min at room temperature. Next, to remove the insoluble debris, the tube was centrifuged at 12,000 g for 10 min at 4 ^°^C; afterward, the supernatant was placed in a microfuge tube. Next, 200 μl chloroform was mixed with the supernatant by inversion for about 30 sec and incubation at room temperature for 2 to 3 min. The tube was put for centrifuging at 12,000 g for 15 min at 4 ^°^C. The upper layer that contained RNA was taken away and poured into another microfuge tube. Next, incubation was applied at -20 ^°^C for 45 min following the addition of isopropanol (0.5 ml) to precipitate the RNA (modified protocol for the maximum RNA precipitation). Following the RNA precipitation, centrifugation was performed at 12,000g for 20 min at 4 ^°^C, and the pellet was washed with 75% ethanol and centrifuged at 7,500 g for 5 min. Finally, the extracted RNA was placed at room temperature to remove ethanol. RNA was mixed in an appropriate volume of DEPC treated water. 1% agarose gel electrophoresis was performed to assess the quality and integrity of isolated RNA. Also, a Nano-Drop UV spectrophotometer (Bio-TeK, USA) was applied to determine the concentration and purity of the extracted RNA. 

***Complementary DNA (cDNA) synthesis***

To synthesize cDNA, Revert Aid First Strand cDNA Synthesis Kit (Fermentas, Burlington, ON, Canada) was used. A total volume of 20 μl, as described briefly below, was used to prepare the necessary mixture for reverse transcription reaction. Following the addition of 1 μl template RNA (5 μg) +1 μl random hexamer primer (15 pmol) into a sterile and nuclease-free tube and adjusting to a volume of 12 μl with DEPC water, incubation was done at 65 ^°^C for 5 min. Subsequently, 4 μl 5X Reaction Buffer, 1 μl RiboLock RNase Inhibitor (20 u/μl), 2 μl 10 mM dNTP Mix, and 1 μl Revert Aid M-MuLV Reverse Transcriptase (200 u/μl) were added to the mixture. The resulting mixture was incubated in a thermocycler device (Eppendorf Master Cycler, USA) for 5 min at 25 ^°^C followed by 60 min at 42 ^°^C. To end the reaction, heating was applied at 70 ^°^C for 5 min (according to the kit manufacturer’s instructions). Agarose gel (1%) electrophoresis was performed for verifying the quality and integrity of the synthesized cDNA. Ultimately, a Nano-Drop UV spectrophotometer (Bio-TeK, USA) was used to specify the purity and concentration of the cDNA.

***Real-time polymerase chain reaction (qPCR)***

The mRNA expression levels of SNARE proteins were amplified and specified using real-time polymerase chain reaction (qPCR) SYBR Premix Ex Taq II (Tli RNaseH Plus) (Takara, China) on CFX96 Real-Time PCR Detection System (Bio-Rad, USA) (23). ). 18s rRNA housekeeping gene was used as an internal control (24). AlleleID7 software (Premier Biosoft Corporation, USA) was used to design gene-specific primers. The primers were prepared at a concentration of 10 µM. Table 1 shows the features of the primer pairs. The qPCR reaction mixture for each sample was made following the kit manufacturer directions as follows: 10 μl SYBR Premix Ex Taq II (1X), 1 μl of each primer (0.4 μM), 1 μl cDNA template (˂100 ng), and 7.0 μl distilled water (20 μl total volume). The reaction was prepared (triplicate), and thermal cycling was done in 40 cycles.

***Insulin assay***

For measuring the serum insulin levels, Rat Insulin (INS) ELISA Kit was applied (based on the kit instruction). Insulin resistance was estimated according to the homeostasis model assessment-estimated insulin resistance (HOMA-IR) index using the following formula: [insulin (μU/ml) × glucose (mmol/l)/22.5] (25).

***Statistical analysis***

Data were expressed as mean±SD. One-way ANOVA, followed by *post hoc* Turkey’s test was employed to compare mean values between different groups (SPSS software, ver. 16). *P*-value< 0.05 was set as significant. 

## Results

During the study period, two rats (one rat in the diabetic control group and one rat in the treated group with RSV dosage of 1 mg/kg body weight) died and thus dropped out from the study.

***Analysis of biochemical parameters***

Biochemical parameters including FBS and body weight were measured at three time periods: at the baseline, the seventh day after diabetes induction (7^th^ day), and at the end of treatment (30^th^ day).

***The effect of RSV on body weight***

 At the beginning of the study, there was no significant difference between the groups in terms of body weight (*P*-value =0.20). After induction of diabetes, body weight significantly decreased in diabetic rats compared to the healthy rats (*P*-value˂0.001) (Table 2). The results indicated that body weight significantly increased in RSV-treated rats compared to diabetic control rats (Table 2). 

***The effect of RSV on serum levels of FBS, insulin, and HOMA-IR index***

Serum levels of FBS were normal at the baseline, and there was no significant difference between groups (*P*-value=0.27). After induction of diabetes, serum FBS levels significantly increased in diabetic rats compared to the healthy rats (*P*-value˂0.001). RSV treatment considerably decreased serum FBS levels in diabetic rats in comparison with the diabetic control rats in which this effect was dependent on the dosage and doses of 5 and 10 mg/kg were more effective than 1 mg/kg (Table 3).

After diabetes induction, the serum insulin levels significantly decreased in diabetic rats compared to the healthy rats (*P*-value<0.001) (Table 3). According to the results, doses of 5 and 10 mg/kg RSV could increase the insulin levels to near normal (*P*-value˂0.001) (Table 3). The results showed that type 2 diabetes was associated with increased HOMA-IR in diabetic rats compared to healthy rats (*P*-value˂0.001) (Table 3). RSV treatment with doses of 5 and 10 mg/kg considerably decreased HOMA-IR in RSV-treated diabetic rats compared to the untreated diabetic rats (*P*-value˂0.05) (Table 3).

***Expression of SNAP-23, syntaxin-4, and VAMP-2 in rat muscle tissue***

Real-time PCR technique was applied to investigate the expression levels of SNAP-23, syntaxin-4, and VAMP-2. The mean delta Ct (cycle threshold) comparisons between experimental groups are reported in Table 3. Gene-specific Ct denotes the number of cycles in which fluorescent signal exceeds the threshold value, and delta Ct is: [interest gene Ct - reference gene Ct]. Lower mean delta Ct shows higher gene expression and vice versa. According to the results, the mean delta Ct of all three genes was lower in diabetic rats compared to that in healthy rats showing higher expression of these genes in diabetic rats; however, the differences were statistically insignificant (Table 4). The mean delta Ct of all three genes in RSV-treated diabetic rats was higher than that in untreated diabetic rats. RSV, in particular at high doses, non-significantly decreased the expression of SNAREs protein genes (Table 4). We calculated the relative fold changes using the 2^- ^^ΔΔCt^ method. The results recommended the up-regulation of the expression of these genes in diabetic rats compared to the healthy rats (syntaxin-4: 9.12-fold, SNAP23:6.06 fold, and VAMP-2: 3.81-fold). The expression of SNAREs protein genes in RSV-treated groups diminished in comparison to the diabetic group (Table 5).

**Table 1:** Properties of primer pairs used in real-time RT-PCR

**SNAP-23**	**SNAP-23**	Syntaxin-4	**VAMP-2**
**NCBI accession number** Sequence (5'->3')	**NM_012663.2**	**NM_022689.2**	**NM_031125.1**
**Forward primer**	CTACTTGGTCCTAAGAATCC	TTCCGTTTCTGTGTCCAATAG	TCAGCAGACTATGTGGAAC
**Reverse primer**	TTGTGCTTTCCAGAGACTCAT	CCAAGATGAGAACAGTGACA	CAGAAGAGTGAAGAGTAATGG

SNAP-23; synaptosomal-associated protein 23, VAMP-2; vesicle-associated membrane protein 2

**Table 2:** The effects of resveratrol (RSV) on body weight in study groups

**Groups**	**Body weight(g)**		
	**Baseline**	**7** ^th^ ** day after diabetes induction**	**End of treatment**
**Cont.**	218.1±17.1	229.3±16.7	260.6±14.7
**Dia.**	220.0±14.1	206.7±14.8 ^a*^	191.2±14.4 ^a*^
**Dia+RSV1**	231.2±18.0	219.38±17.0 ^a^	222.6±17.6^b^
**Dia+RSV5**	228.0±15.0	215.8±7.61 ^a^	221.5±14.9^b^
**Dia+RSV10**	210.3±18.3	203.6±17.0 ^a*^	208.8±27.6^b^

Result are represented as mean ±SD

Cont: Healthy control group, Dia.: diabetic control group, Dia+RSV1, Dia+RSV5, and Dia+RSV10: diabetic rats treated with doses of 1, 5, and 10 mg/kg bwt of RSV, respectively

^a^: *P-value<*0.05 comparing with healthy control group. ^a^*: *P-value<*0.001 comparing with healthy control group

^b^: *P-value <*0.01 comparing with diabetic control

**Table 3:** The effects of resveratrol (RSV) on serum levels of Fasting blood sugar (FBS), insulin, and Homeostatic Model Assessment of Insulin Resistance (HOMA-IR)

**Groups**	**FBS(mg/dL)**			**Insulin** **(** **μU/ml** **)**	**HOMA-IR**
	**Baseline**	**7** ^th^ ** day after diabetes induction**	**End of treatment**		
**Cont.**	87.3±10.6	89.12±10.7	92.0±10.8	11.17±1.06	2.51±0.37
**Dia.**	95.62±7.3	279.2±95.3^a^	303.3±92.1^a^	7.23±1.15^a^	5.46±2.09^a^
**Dia+RSV1**	93.2±14.8	289.7±79.2^a^	271.3±77.5	8.13±0.97	5.52±1.92
**Dia+RSV5**	84.1±11.0	219.3±120^a^	192.5±84.8^b^	9.52±1.0.5^b^	3.73±2.01^c^
**Dia+RSV10**	90.5±7.23	241.2±99.8^a^	190.3±68.6^b^	9.83±0.86^b^	4.77±1.08^c^

Results are represented as mean ±SD

FBS: Fasting blood sugar; HOMA-IR: Homeostatic Model Assessment of Insulin Resistance. Cont.: Healthy control group, Dia.: diabetic control group, Dia+RSV1, Dia+RSV5, and Dia+RSV10 are diabetic rats treated with doses of 1, 5, and 10mg/kg body weight of RSV, respectively

^a^: *P-value <*0.001 compared with the control group (Cont.). ^b^: *P-value<*0.001 compared with the diabetic group (Dia.). ^c^: *P-value<*0.05 compared with the diabetic group

**Table 4:** The effect of resveratrol (RSV) on expression of SNAP-23, Syntaxin-4, and VAMP-2 (ΔCt Value)

Dia + RSV10	Dia + RSV5	Dia + RSV1	Dia.	Cont.	
10.31 ±4.83	9.68 ±2.86	9.38 ±3.11	7.47± 3.23	10.07±1.57	SNAP-23
9.00 ± 1.70	9.48 ± 2.44	8.56 ±3.94	6.75±1.78	9.94 ± 3.01	Stx4
10.90± 2.22	9.07 ± 1.47	9.00 ±1.70	8.45± 2.90	10.83 ±2.06	**Vamp-2 **

Results are represented as mean ±SD.

Cont.: Healthy control group, Dia.: diabetic control group, Dia+RSV1, Dia+RSV5, and Dia+RSV10 are diabetic rats treated with doses of 1, 5, and 10mg/kg body weight of RSV, respectively

SNAP-23; synaptosomal-associated protein 23, Stx4; Syntaxin 4, VAMP-2; vesicle-associated membrane protein 2

**Table 5.** Fold change (2- ^ΔΔCt^) of SNARE proteins expression in studied groups

Groups	SNAP-23	Syntaxin-4	Vamp-2
Dia. - Cont.	6.06	9.12	3.81
(Dia + RSV1) - Dia.	0.26	0.28	0.68
(Dia + RSV5) - Dia.	0.21	0.15	0.65
(Dia + RSV10) - Dia.	0.13	0.21	0.18

ΔΔCt: ΔCt [case-control]

Cont.: Healthy control group, Dia.: diabetic control group, Dia+RSV1, Dia+RSV5, and Dia+RSV10 are diabetic rats treated with doses of 1, 5, and 10mg/kg body weight of RSV, respectively

## Discussion

Since impaired insulin secretion and insulin resistance are two leading pathological causes of T2DM, identification of effective molecular mechanisms in these events is necessary for the prevention and treatment of T2DM. GLUT4 is exclusively expressed in adipose tissue and skeletal muscles. Insulin-stimulated delivery of GLUT4 to the muscle cell surface is mediated by SNARE complex proteins. Among different isoforms of SNARE proteins, SNAP-23, VAMP-2, and Syntaxin-4 are involved significantly in the transfer of GLUT4 and glucose uptake in muscle cells (26). Since skeletal muscles are responsible for around 80% of glucose uptake, defects in GLUT4 traffic lead to insulin resistance and hyperglycemia (27). The present study explored the impact of RSV as an antioxidant with anti-diabetic effect on the SNARE proteins expression in T2DM. In this study, T2DM was induced by STZ-NA injection. Nicotinamide exerts antioxidant effects and partially protects the beta cells against STZ-damage by preventing the activity of the poly (ADP-ribose) polymerase and maintaining intracellular ATP and NAD^+^ ; therefore, it has been demonstrated as an appropriate approach to make a model for T2DM (28). Various studies have reported the influence of RSV on diabetes through multiple mechanisms. It is proven that chronic administration of RSV improves hyperglycemia and treats the symptoms of T2DM, and also prevents weight loss in STZ-NA-induced diabetic rats (17). Consistent with these findings, the present study reported that administrating RSV orally, considerably decreased weight loss and blood glucose in RSV treated diabetic rats. These effects were dependent on dosage, and doses of 5 and 10 mg/kg were more effective. Some studies have shown that insulin levels increased in response to RSV (10 mg/kg) and improved insulin sensitivity in STZ-NA induced diabetic rats (29). Our findings demonstrated that RSV elevated the insulin levels and reduced HOMA index in RSV-treated rats. It has been shown that RSV binds to the sulfonylurea receptors, in which there is a link between the stimulation and activation of these receptors with blockade of ATP-sensitive K^+^ channels leading to depolarization of the β cell membrane and insulin release (26, 28). It is believed that Sirtuin 1 protein (SIRT1) activation is the key mechanism of RSV on glucose homeostasis and insulin sensitivity (30). Some studies have reported that RSV enhances GLUT4 translocation to the caveolar lipid raft fractions through AMPK/Akt/eNOS signaling pathway in the diabetic myocardium (16, 17). It has also been shown that RSV is able to reduce the production of reactive oxygen species (ROS) by activating antioxidant enzymes and inhibiting pro-oxidant enzymes which can increase insulin sensitivity (31). 

SNARE protein abnormalities have been reported in several studies. Studies on Goto-Kakizaki (GK) rats with moderate hyperglycemia and insulin resistance and *fa/fa *Zucker rats have shown that expression of different isoforms of the SNARE proteins was significantly reduced in pancreatic β cells (11, 32). In contrast, some studies have shown that SNARE protein levels could increase in skeletal muscles under conditions of hyperglycemia and hyperinsulinemia (12, 33). According to the studies, GLUT4 content and insulin levels are the effective factors in SNARE protein levels.

Maier *et al.* explored variations in SNARE protein levels in Zucker diabetic fatty (ZDF) rats with hyperglycemia/hyperinsulinemia and concurrently in STZ-induced diabetic rats with hyperglycemia in the absence of hyperinsulinemia (12). The results showed a significant rise in SNARE protein levels in skeletal muscles of Zucker rats without changes in GLUT4 levels, while GLUT4 content significantly diminished in STZ-induced hyperglycemic rats without any changes in the SNARE protein levels compared with the lean controls (12). In this study, rosiglitazone normalized the levels of SNARE protein in Zucker rats by improving insulin sensitivity. Another study found that GLUT4 levels increased in response to RSV treatment in the soleus muscle of STZ-diabetic rats (29). Our findings, in accordance with previous studies, showed changes in the expression of SNARE proteins in skeletal muscles of diabetic rats compared to healthy control rats, where the expressions of SNAP-23, VAMP-2, and Syntaxin-4 were non-significantly enhanced. This growth in the expression of SNARE complex proteins was probably a compensatory mechanism in mild hyperglycemia and insulin resistance conditions; nevertheless, in this study, the GLUT4 content was not evaluated in the skeletal muscles. Also, the previous reports have suggested that the expression of Munc 18c protein increased under insulin resistance conditions. Munc 18c is a regulatory protein for SNARE complex proteins which binds to Syntaxin-4 and prevents the formation of a ternary complex and transfer of GLUT4 to the cell surface (34). Under these conditions, the expression of SNARE complex proteins possibly increased as a compensatory mechanism in parallel with enhanced expression of this inhibitory protein. This, in turn, can be a reason for the elevated expression of these proteins in T2DM. In particular, our results showed that Syntaxin-4 had the highest expression compared to VAMP-2 and SNAP-23. In addition, the expression of SNAP-23 increased more than VAMP-2, maybe because of the catalytic role of SNAP-23 to form a ternary complex of SNARE proteins. It is also known that nicotinamide prevents AMPK activation by inhibiting SIRT1 phosphorylation, which can lead to insulin resistance and reduced GLUT4 (29, 35); therefore, it is likely that RSV has modified the expression of SNARE proteins through increased GLUT4 transport to the muscle cell surface by activating SIRT1 and improving insulin sensitivity. Gene expression analysis revealed that RVS, especially at high doses, reduced the expression of SNARE complex proteins; however, this reduction was not statistically significant. According to various animal studies, the most effective therapeutic doses of RSV are daily dosages of 5–100 mg/kg body weight; therefore, it seems that taking higher doses of RSV or a longer period of intervention can be more effective. 

Our team also studied the influence of RSV on SNARE complex proteins in adipose tissue of diabetic rats; remarkably, the expression of SNARE complex proteins in diabetic rats was considerably decreased compared to healthy rats; RSV was capable of modifying the expression of SNARE complex proteins to normal levels. (36). The results of a study suggested that the SNARE-regulating protein Munc18c expression in skeletal muscles was enhanced in patients with type 2 diabetes; while no change was found in the expression of Munc18c in adipose tissue (33). This difference in the expression of Munc18c in skeletal muscles and adipose tissue may be one of the causes of different outcomes in the expression of SNARE complex proteins in these tissues, though further research is required.

One of the strengths of this study was exploring the impact of RSV as a natural polyphenol, having antioxidant properties, with anti-diabetic features on the expression of SNARE proteins in skeletal muscles of STZ-NA-induced diabetic rats for the first time. It is suggested to evaluate the effect of RSV on the levels of SNARE complex proteins and GLUT4, which can be mentioned as a limitation of our study. Our team also evaluated the effects of RSV on the expression of adipokines secreted from adipose tissue. The results indicated that RSV improved insulin sensitivity by reducing inflammatory adipokines and elevating anti-inflammatory adipokines (37, 38). 

## Conclusion

In the current study, there was a link between the induced diabetes with insulin resistance and up-regulation of SNARE proteins expression in skeletal muscles. RSV non-significantly reduced the expression of these genes. This effect was dose-dependent, so it was likely that higher doses of RSV and longer periods of intervention would be more effective. Our findings shed light on another molecular mechanism for anti-diabetic features of RSV that influence SNARE proteins expression.
